# Mechanochemical preparation of chrysomycin A self-micelle solid dispersion with improved solubility and enhanced oral bioavailability

**DOI:** 10.1186/s12951-021-00911-7

**Published:** 2021-05-31

**Authors:** Zhuomin Xu, Shanshan Zheng, Xin Gao, Yulu Hong, Yue Cai, Qiuqin Zhang, Jiani Xiang, Dehui Xie, Fuxing Song, Huawei Zhang, Hong Wang, Xuanrong Sun

**Affiliations:** 1 Collaborative Innovation Center of Yangtze River Delta Region Green Pharmaceuticals and College of Pharmaceutical Science, Zhejiang University of Technology, Hangzhou, 310014 China; 2 School of Light Industry, Beijing Technology and Business University, Beijing, 100048 China

**Keywords:** Chrysomycin A, Mechanochemistry, Ball milling, Solid dispersion, Self-micelle, Bioavailability, Antitumor

## Abstract

**Background:**

Chrysomycin A (CA) has been reported as numerous excellent biological activities, such as antineoplastic and antibacterial. Though, poor solubility of CA limited its application in medical field. Due to good amphiphilicity and potential anticancer effect of disodium glycyrrhizin (Na_2_GA) as an excipient, an amorphous solid dispersion (Na_2_GA/CA-BM) consisting of CA and Na_2_GA was prepared in the present study by mechanochemical technology (roll mill ML-007, zirconium balls, 30 rpm, 2.5 h) to improve the solubility and oral bioavailability of CA. Then, Na_2_GA/CA-BM was self-assembled to micelles in water. The interaction of CA and Na_2_GA in solid state were investigated by X-ray diffraction studies, polarized light microscopy, and scanning electron microscope. Meanwhile, the properties of the sample solution were analyzed by dynamic light scattering and transmission electron. Furthermore, the oral bioavailability and antitumor ability of Na_2_GA/CA-BM in vivo were tested, providing a theoretical basis for future application of CA on cancer therapy.

**Results:**

CA encapsulated by Na_2_GA was self-assembled to nano-micelles in water. The average diameter of nano-micelle was 131.6 nm, and zeta potential was − 11.7 mV. Three physicochemical detections showed that CA was transformed from crystal into amorphous form after treated with ball milling and the solubility increased by 50 times. Na2GA/CA-BM showed a significant increase of the bioavailability about two time that of free CA. Compared with free CA, the in-vivo antitumor studies also exhibited that Na_2_GA/CA-BM had an excellent inhibition of tumor growth.

**Conclusions:**

Na_2_GA/CA-BM nanoparticles (131.6 nm, − 11.7 mV) prepared by simple and low-cost mechanochemical technology can improve oral bioavailability and antitumor efficacy of CA in vivo, suggesting a potential formulation for efficient anticancer treatment.

**Graphic abstract:**

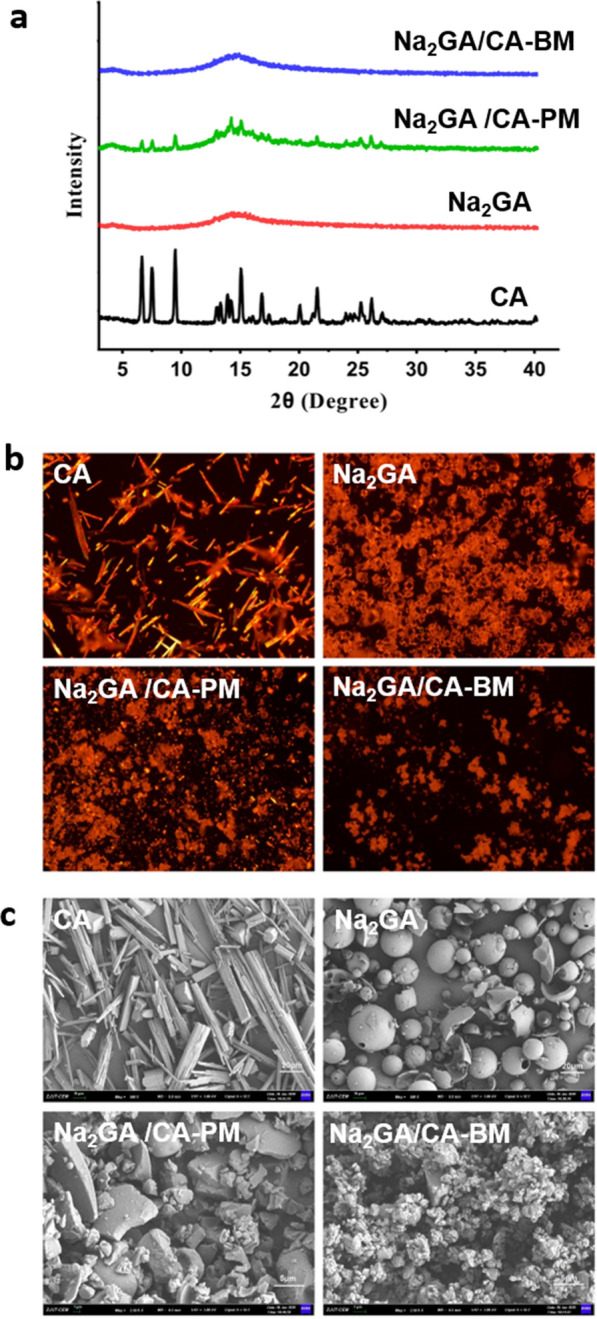

## Introduction

**Fig. 1:**
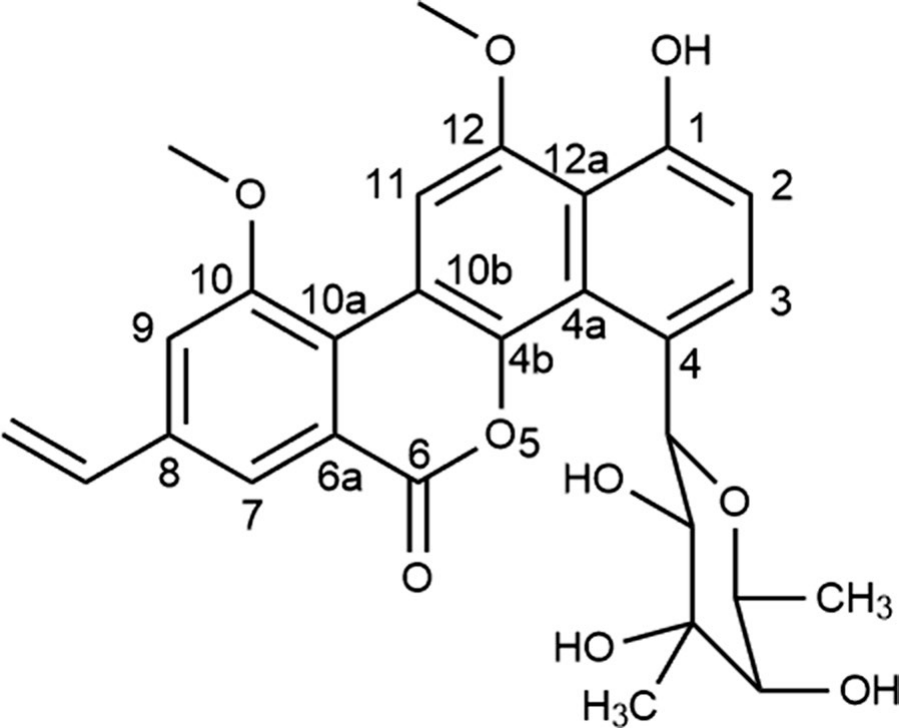
Chemical structure of chrysomycin A

Chrysomycins is a novel antibiotic complex isolated from *Streptomyces* spp, containing compounds of C-glycosides antitumor actives [1]. Especially, Chrysomycin A (CA, Fig. 1) is the major analogue of chrysomycins and plays the most potent role in this complex [2]. Compared with the clinically used anticancer agent doxorubicin, CA shows significant cytotoxicity toward cancer cells because of its vinyl group in the 8-position [3, 4]. In addition to strong antineoplastic and antibacterial properties of CA [5–7], it is thought to act as an inhibitor of the catalytic activity of human topoisomerase II [8]. Besides, CA equips with strong antifungal profile, and its cytotoxicity to normal cells can be negligible [2]. Meanwhile, it has no effect on the lysis of red blood cells [6]. All these characteristics indicate that CA has the potential to be a good anti-tumor, anti-bacterial and even anti-fungal candidate. Nonetheless, the oral bioavailability of CA is low owe to its poor solubility in water, which restricts its clinical application. To the best of our knowledge, there are no studies on how to overcome these shortcomings of CA.

Generally, several approaches were employed to improve drug insolubility and bioavailability, such as preparation of polymeric micelles [9, 10], cyclodextrins inclusion complex [11, 12], solid dispersions (SDs) [13, 14], self-emulsifying drug delivery system [15], and so on. In the methods mentioned above, most of them need multiple organic solvents (e.g., dimethyl sulfoxide, N,N-dimethylformamide, dichloromethane, etc.), large quantity of surfactants, complex procedures, long preparation time, or expensive excipients (including cholesterol, lipids) [16]. Those are considered unfriendly to the environment, and may increase the risk of solvent exposure during preparation and the cost of production.

Mechanochemical technology has become extensively popular in the field of pharmaceutical sciences for its important role in the development of green synthesis [17, 18], cocrystal synthesis [19, 20], and amorphous SDs [21, 22]. When the high intensity of mechanical energy is transferred to the solid state substances, the strain is generated and may cause plastic deformation and concurrent changes in the crystal structure along with crystalline phase transitions and amorphization [23]. All the changes may potently enhance the solubility and bioavailability [24]. Compared with traditional “liquid phase” way, mechanochemical treatment provides significant advantages such as one-step technological process, absence of solvents, and low operating cost.

Disodium glycyrrhizinate (Na_2_GA) is the salt formation of glycyrrhizic acid (GA), which can undergo hydrolysis in aqueous solutions and generated free GA. As for GA, it is a good soluble natural saponin, having antiviral [25], anti-inflammatory [26] and anticancer [27] properties. Apart from those features, GA forms non-covalent compounds with various drugs due to its amphiphilicity. Such supramolecular compounds could increase the solubility of hydrophobic drugs up to dozens of times, and enhance the permeability of drug through cell membranes [28–30]. In contrast, Na_2_GA solution has lower viscosity and more environment-friendly than GA solution. Meanwhile, Na_2_GA has also been reported to have antitumor activity. Zhang et al. [16] formed an amorphous SD of curcumin and Na_2_GA utilizing mechanochemistry to enhance the bioavailability and cytotoxic activity of curcumin. Zhu et al. [22] encapsulated SN-38 into Na_2_GA for preparing a SN-38 self-micelle SD resulting in markly improving the solubility and antitumor activity of SN-38.

Given that mechanochemical technology and Na_2_GA have the potential to improve the solubility and bioavailability of water insoluble drug, in this study, solid dispersion of CA were prepared mechanical milling with Na_2_GA. The physical characteristics, solubility, pharmacokinetics, tissue distribution and anti-tumor activity of CA as an amorphous SD were further investigated.

## Materials and methods

### Materials

CA was obtained from professor Fuxing Song (Beijing Technology and Business University, purity > 99%). Disodium salt of glycyrrhizic acid (Na_2_GA) was purchased from Shanxi Pioneer Biotech Co. Ltd. (Xian, China, purity > 98%). Acetonitrile was obtained from Tedia Company, Inc. (Fairfield, OH, USA, HPLC grade). Formic acid with purity > 88% was purchased from Aladdin Bio-Chem Technology Co., Ltd. (Shanghai, China). Roswell Park Memorial Institute 1640 (RPMI-1640) cell culture medium, fetal bovine serum (FBS) and penicillin/ streptomycin were all purchased from Gibco BRL (Gaithersburg, MD, USA).

### Cells and animals

The mouse melanoma cell line B16-F10, the human breast cancer cell line MCF-7, the human hepatocellular carcinoma cell line HepG_2_ was purchased from the China Center for Type Culture Collection (Wuhan, China). The cells were cultured in RPMI-1640 (B16-F10 cells) or DMEM (MCF-7 cells and HepG2 cells) containing 10% FBS and 1% antibiotics (penicillin/streptomycin).

Female ICR mice (5–6 weeks of age, 18–20 g body weight) and female C57BL/6 mice (5–6 weeks of age, 16–18 g) used in the experiments were provided by the Zhejiang Academy of medical Science, conducting with the approval of the animal experiment center of Zhejiang University of Technology. All the animals were performed in strict compliance with the PR China legislation for the use and care of laboratory animals.

### Fabrication of nanoparticles by mechanochemical treatment

The roll mill ML-007 (Wiggens, German) was used to prepare samples. Briefly, 0.15 g CA and 14.85 g Na_2_GA (weight ratio 1/99) were added to 300 mL vial with 660.0 g zirconium balls (diameter 22 mm) with milling time of 3 h, rotation speed 30 rpm and samples were picked out at 0.5, 1, 1.5, 2, 2.5 h, and 3 h, respectively. In addition, a mixture of Na_2_GA and CA (weight ratio was same as above) by ordinary physical treating without ball milling, were prepared for comparing with the ball milling products. At last, the ball-milling products with different milling time were described as BM-0.5 h, BM-1.0 h, BM-1.5 h, BM-2.0 h, BM-2.5 h, BM-3 h, and the physical milling product was described as Na_2_GA/CA-PM.

### Analysis of chrysomycin A by HPLC

The appropriate amounts of samples were dissolved completely in a mixture solution (deionized water to acetonitrile, 1:1, v/v) respectively, and filtered through a 0.22 μm filter paper. Then, the filterate was determined by a high performance liquid chromatography (HPLC, Aglient 1260 infinity II) equipped with column Inertisil O DS-3 C_18_ (250 mm × 4.6 mm, 5 μm, GL Science Inc., Japan) at 25 ℃, and a UV detector set at a wavelength of 254 nm. Acetonitrile—0.1% formate water (40:60) was used as eluent (pH = 2.6–2.8) with the flow rate of 1.0 mL/min.

### Solubility determination

To determine the solubility, an overdose of samples and CA, were put into 500 μL of deionized water respectively and stirred for 12 h at 25 °C. Finally, these solutions were filtered and analyzed by HPLC.

### Powder X-ray diffraction (XRD)

X-ray diffraction test of samples was implemented with a Bruker D_2_ Phase diffractometer (Buker, Germany) by using CuKα radiation. Step range: 3°–40°. Counter speed: 3.7°/min. All the data were analyzed through GraphPad Prism 7.

### Polarized light microscopy (PLM)

To distinguish the refraction phenomenon of samples, a small amount of solid powder was placed on microscope slide and observed by an Olympus CX41 polarized microscope (Japan) with a CCD camera (HTC1600, China). All the pictures were obtained at 10× resolution.

### Scanning electron microscopy (SEM)

After samples were coated with platinum by a Leica EM ACE200 Vacuum Coater (Germany), SEM (ZEISS Gemini500, Germany) was performed to acquire electronic images. The Coating parameter: amperage 30 mA, spraying time 100 s.

### Particle characterization, zeta potential and stability

The physicochemical properties of samples containing hydrodynamic diameter, polydispersity index (PDI), and zeta potential, were detected using dynamic light scattering (DLS) instrument (Zetasizer NanoZS, Malvern Instruments, Malvern, UK) at 25 °C. Before being measured, all samples were dissolved in deionized water at the concentration of 1 mg/mL, then filtered by a 0.22 μm filter. The sample was dissolved in DMEM with 10% FBS. Then the change in particle size and PDI of micelles were measured for 72 h.

### Determination of the critical micelle concentration

3 μg of Nile red dissolved in 90 μL CH_2_Cl_2_ was added to a series pf vials, and CH_2_Cl_2_ was evaporated at room temperature. The aqueous solutions of ball milling sample with various concentrations ranging from 0.001 to 10 mg/mL were added into the vials, stirred for 12 h. The fluorescence intensity of Nile red (excitation wavelength: 579 nm, emission wavelength: 620 nm) in these solutions was measured by a microplate reader (Flexstation 3, Molecular Devices LLC, Sunnyvale, CA, USA).

### Transmission electron microscopy (TEM)

To observe the morphology of micelle, samples were configured into 1 mg/ml solution. One drop of sample was dripped on a carbon Formvar-coated cooper grid for a minute, and then were dried below the infrared light. Finally, TEM (Hitachi HT700 EXALENS, Japan) was at a working voltage of 100 kV to form the morphology of samples.

### In vitro cell viability studies

Cell viability of free CA, Na_2_GA, and the ball milling sample on MCF-7, HepG_2_, and B16-F10 cells was evaluated by MTT assay. The cells were incubated in 96-well plates at a density of 4 × 10^3^ cells per well. After 12 h of incubation at 37 °C with 5% CO_2_, the medium was replaced by 100 μL fresh medium containing the suspension of free CA, Na_2_GA, or the ball milling sample with a series of concentration. After another 48 h of incubation, the medium was removed and the fresh medium containing 10 μL of MTT (5 mg/mL) were added to each well. The cells were further incubated for 4 h, then the medium was removed, and 100 μL DMSO was added to dissolve the formazan crystals. The absorbance of each wells was measured be a microplate reader at the wavelength of 570 nm. Cell viability in each group was expressed as a percentage relative to that of the untreated control.

### Cellular uptake studies

For qualitative analysis and intracellular localization, coumarin-6-loaded ball milling products (NPs/C6) were prepared. 1 mg coumarin-6, were mixed with 1 mg Na_2_GA/CA-BM powders and then dissolved in 200 μL tetrahydrofuran completely. Then about 1 mL distilled water was added dropwise with continuously stirring for extra 12 h. When tetrahydrofuran was evaporated, the labeled NPs were stored at − 20 °C.

B16-F10 cells were seeded in a 24-well plate as a density of 2 × 10^4^ cells per well, and incubated for 12 h before use. Then the cells were incubated with 10 μg/mL NPs/C6. Four hours later, the cells were washed three times with 4 °C PBS, fixed with 4% paraformaldehyde for 15 min at room temperature, and stained with Hoechst33342 for another 10 min. Finally, the plate was observed under a fluorescence microscope (Olympus IX73, Japan) after washed with 4 °C PBS three times.

### Pharmacokinetic evaluation

Ten female ICR mice were randomly divided into two groups (CA and the ball-milling produc to evaluate the pharmacokinetic of samples. The samples were dispersed in deionized water and were intragastriclly administered to the mice at the equivalent dose of 50 mg/kg CA. Next, 0.2 mL of blood was collected into prepared heparinized tubes at different time points (0.25, 0.5, 1, 2, 4, 8, 12, and 24 h) after administration, and then centrifuged at 5000 rpm, 4 °C for 5 min to obtain plasma supernatant. After taking plasma to a cleaning tube, a certain volume of acetonitrile was added to the supernatant (the volume ratio was 3:1). When protein precipitates generated, the mixture was vortexed for 2 min, and centrifugated at 10,000 rpm, 4 °C for 10 min. Then, supernatant from the mixture was extracted and stored at − 80 °C for 2 h for furth use. After being thawed, samples were centrifugated (10,000 rpm, 4 °C) for 10 min and take out. At last, the sample was filtered by a 0.22 μm filter for HPLC analysis.

### In vivo tissue biodistribution study

To investigate the tissue biodistribution of CA and the ball-milling product, ten female ICR mice were stochastically divided into two groups. The ball-milling sample and CA were formulated as suspensions at a concentration of 5 mg/mL. The dose for each intragastric administration was 50 mg/kg equivalent to the concentration of CA. At the set time points (2 h, 6 h, 12 h), major organs containing heart, liver, spleen, lung, kidney, brain, skeletal muscle were resected and wash with 10 mM phosphate buffered saline (PBS). After being dried and weighted, the organs were divided into small pieces and homogenized with deionized water at ratio of 1:2 (g/mL). To extract CA from tissues, the homogenate was added with acetonitrile (the ratio was 1:3). Then the mixture was vortexed for 1 min and centrifuged at 10,000 rpm, 4 °C for 10 min. Ultimately, supernatant was removed from the mixture to a clean tube and stored at − 80 °C for HPLC analysis.

### In vivo antitumor efficacy

The tumor-bearing model was established by subcutaneously injecting 1 × 10^6^ B16-F10 cells in 100μL of PBS into female C57BL/6 mice at the right flank. When the tumor volume reached to about 35–60 mm^3^, the mice were casually divided into three groups (n = 6/group). Each mouse was intragastrically administered with an equivalent dose of 50 mg/kg CA (Na_2_GA/CA-BM and CA suspension) every 2–3 days, whereas the control group was given PBS. The volume of administration was 200 µL per mouse.

The first day of administration was recorded as day 0, the tumor growth and body weight change were monitored every 2–3 days. The tumor volume was measured with a caliper and was calculated as follows: tumor volume = 0.5 × length × width^2^. On the 12th day, the mice were sacrificed, then the tumor and major organs (hearts, lungs, livers, kidneys and spleens) were washed with PBS and weighed. Moreover, tumor paraffin sections of three groups were stained with H&E staining to observe pathological changes.

### Statistical analysis

Data were reported as mean ± standard error of the mean, using the unpaired Student’s *t*-test. Values of *p < 0.05 and ***p < 0.001 calculated by GraphPad Prism 7 were considered significant and extremely significant, respectively.

## Results and discussion

### Solubility determination of chrysomycin A SDs

The aqueous solubility of CA and its ball milling products was shown in Table 1. It could be seen that there were significant differences between CA (1.68 ± 0.66 μg/mL) and the SD samples. In addition, the solubility of CA SDs was gradually increased by prolonging the ball milling time from 0.5 to 2.5 h. The drug milled for long time provided better wettability and dispersibility which was formed as the amorphous complex and encapsulated in a hydrophilic carrier. The solubility of Na_2_GA/CA SD was excellently raised after the formation. However, an unwanting decreased of the solubility could be observed after milling for 3 h. It was supposed that further aggregation of the particles resulted in their higher surface energy with increased time of milling process, and thereby decreasing the solubility [31]. Since the sample created by ball milling for 2.5 h had the best solubility (82.41 ± 25.32 μg/mL) which was increased about 50 times compared with unprocessed pure CA, it was chose as chrysomycin A SDs candidate to study the subsequent experiments and was described as Na_2_GA/CA-BM.

**Table 1:** The solubility of pure CA and its mechanical processed products

Sample	Ball-milling time (h)	Solubility (μg/mL)	Increase solubility (times)
CA	–	1.68 ± 0.66	–
BM-0.5 h	0.5	38.39 ± 8.86	~ 23
BM-1.0 h	1	50.97 ± 27.89	~ 30
BM-1.5 h	1.5	56.21 ± 16.89	~ 33
BM-2.0 h	2	63.78 ± 11.53	~ 38
BM-2.5 h	2.5	82.40 ± 25.32	~ 50
BM-3.0 h	3	65.23 ± 17.16	~ 39

### Physicochemical changes of chrysomycin A SDs

**Fig. 2:**
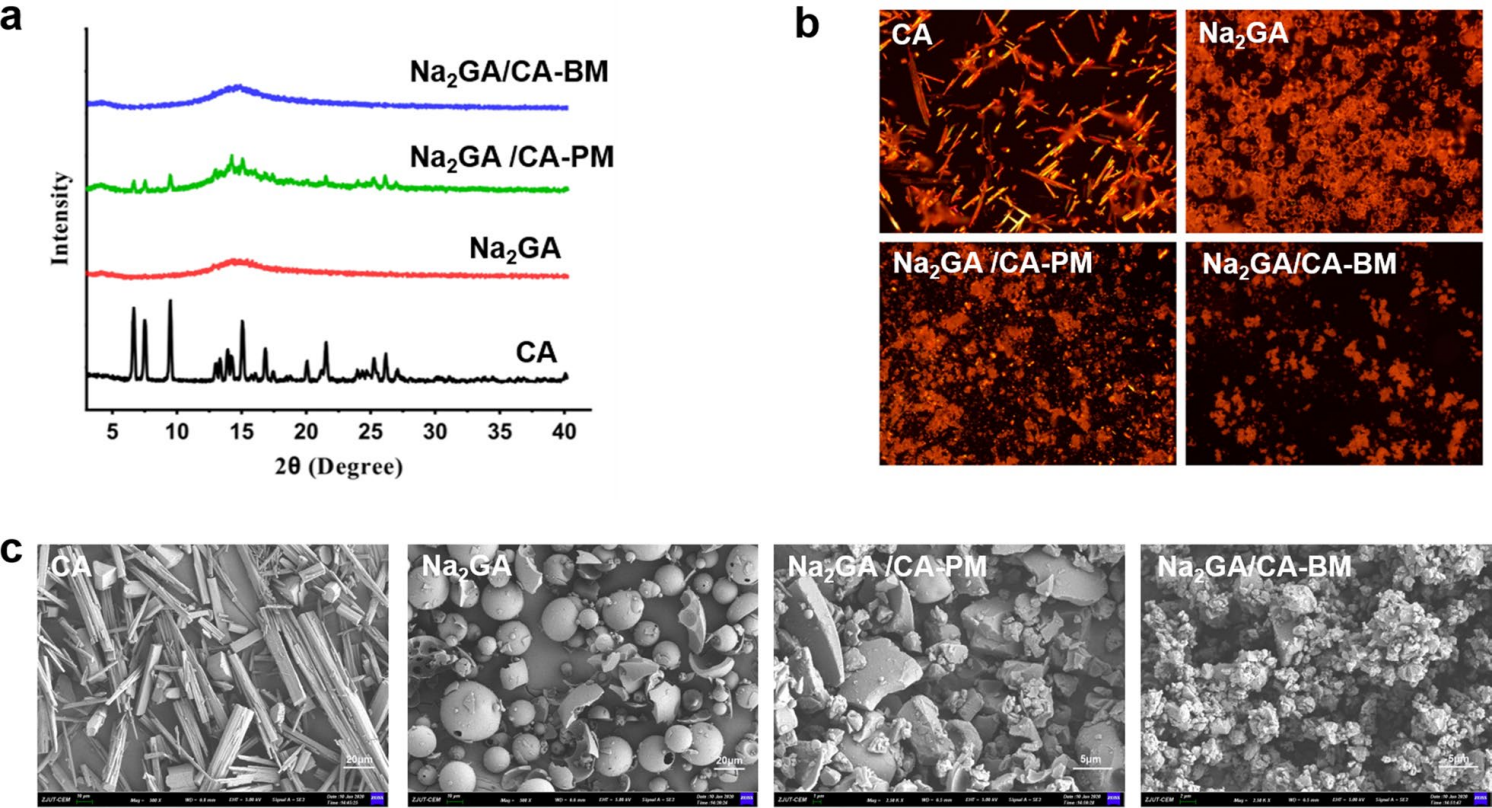
**a** X-ray diffraction spectra of CA, Na_2_GA, Na_2_GA/CA-PM, Na_2_GA/CA-BM. **b** PLM images of CA, Na_2_GA, Na_2_GA/CA-PM, Na_2_GA/CA-BM, the magnification was 10×. **c** The electron micrographs of CA, Na_2_GA, Na_2_GA/CA-PM, Na_2_GA/CA-BM (The scale bars of **CA**, **NA**_**2**_**GA** were 20 μm, the scale bars of **NA**_**2**_**GA/CA-PM**, **NA**_**2**_**GA/CA-BM** were 5 μm)

Physicochemical changes were analyzed by XRD, PLM and SEM. The X-ray diffractograms of CA, Na_2_GA, Na_2_GA/CA-Pand Na_2_GA/CA-BM were shown in Fig. 2a. CA displayed several sharp peaks at diffraction angles (2θ) of 6.67, 7.50, 9.47, 15.09, 21.60, indicating its crystalline form. On the other hand, the characteristic peaks of CA existed in the mechanical treated sample indicating it was still a crystal form. However, the crystallization peaks of CA were markedly decreased in the diffraction spectrum of Na_2_GA/CA-PM, and even no characteristic peaks were observed in the sample of Na_2_GA/CA-BM. The phenomenon could be attributed to the completely loss of crystalline of CA owing to high-intensity ball milling process. These XRD results further confirmed that CA which was dispersed in excipient Na_2_GA to form an amorphous complex by ball milling.

The micrographs of CA, Na_2_GA, Na_2_GA/CA-PM and Na_2_GA/CA-BM obtained from polarized light microscopy are shown in Fig. 2b. As observed in the unprocessed CA, there was extensive birefringence, confirming its crystalline nature. In the Na_2_GA/CA-PM micrograph of the physical mixture, the birefringence of CA was dispersed partially. After mechanochemical treatment, Na_2_GA/CA-BM was shown no birefringence which identified the amorphous nature of CA embedded in Na_2_GA.

Furthermore, the electron micrographs of CA, Na_2_GA, Na_2_GA/CA-PM and Na_2_GA/CA-BM are shown in Fig. 2c. It could be clearly seen that pure CA was elongated solid and the Na_2_GA was composed of hollow spherical particle with a smooth surface texture. On the contrary, the intact morphology of CA and Na_2_GA were disappeared and showed a fine and irregularly shaped particle for the ball-milling product Na_2_GA/CA-BM, suggesting the amorphous solid phase structure of Na_2_GA/CA-BM. The most noteworthy, that the noted particles dispersed more uniformly after being ground for 2.5 h, possibly increased its surface thus improving the velocity of dissolution.

### Properties of chrysomycin A micelles in water solution

**Fig. 3:**
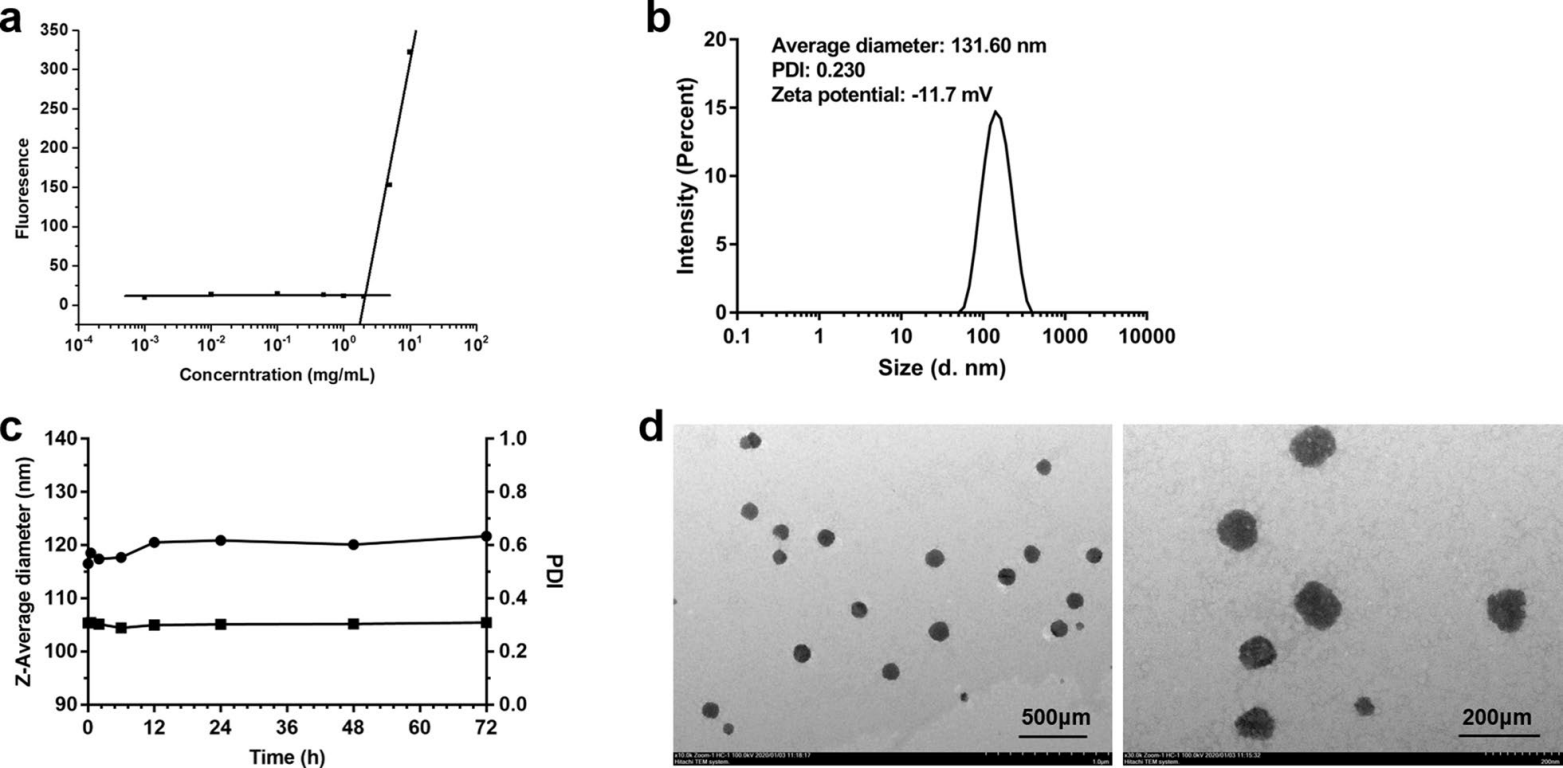
The CMC, size, zeta potential and surface morphology of Na_2_GA/CA-BM. **a** The CMC value of Na_2_GA/CA-BM. **b** Dynamic light scattering size measurement of Na_2_GA/CA micelles. **c** The stability of Na_2_GA/CA-BM. **d** Transmission electron micrograph (TEM) of Na_2_GA/CA micelles, the scale bars from left to right were 500 μm, 200 μm

When the Na_2_GA/CA-BM dissolved in water, Na_2_GA coated CA to form CA micelles. The critical micelle concentration of Na_2_GA/CA-BM was about 1.77 mg/mL (Fig. 3a). The size, zeta potential and surface morphology of micelles are all crucial for interactions between the cell membranes and micelles. As shown in Fig. 3b, the average diameter of the particle was about 131.6 nm with a narrow size distribution at 25 °C, and its polymer dispersity index (PDI) value was about 0.230. Moreover, the particle has a negative zeta potential which was − 11.7 mV. It was reported that suitable range of particle sizes for evading filtration in reticuloendothelial system (RES) organs was between 100 and 200 nm [32–35]. In addition, the neutral surface charge of particles (zeta potential ± 10 mV) was proved to prolonged blood circulation and facilitate its accumulation at the tumor tissue [33]. Therefore, Na_2_GA/CA-BM formed a great candiate to further use in the animal studies due to proper diameter and potential. The particle size showed relatively stable over a span of 72 h incubation in cell culture medium with10% FBS and a slight increase of size from 116 to 121 nm during the period. The PDI remained relatively the same at about 0.30. Furthermore, the images of micelle appearance observed by TEM are depicted in Fig. 3d. The nano-micelle was spherical with smooth boundaries. The diameter of nano-micelle was about 100 nm and slightly smaller than DLS data because of its shrinkage when dried before TEM detection.

### In vitro cytotoxicity and cell uptake

**Fig. 4:**
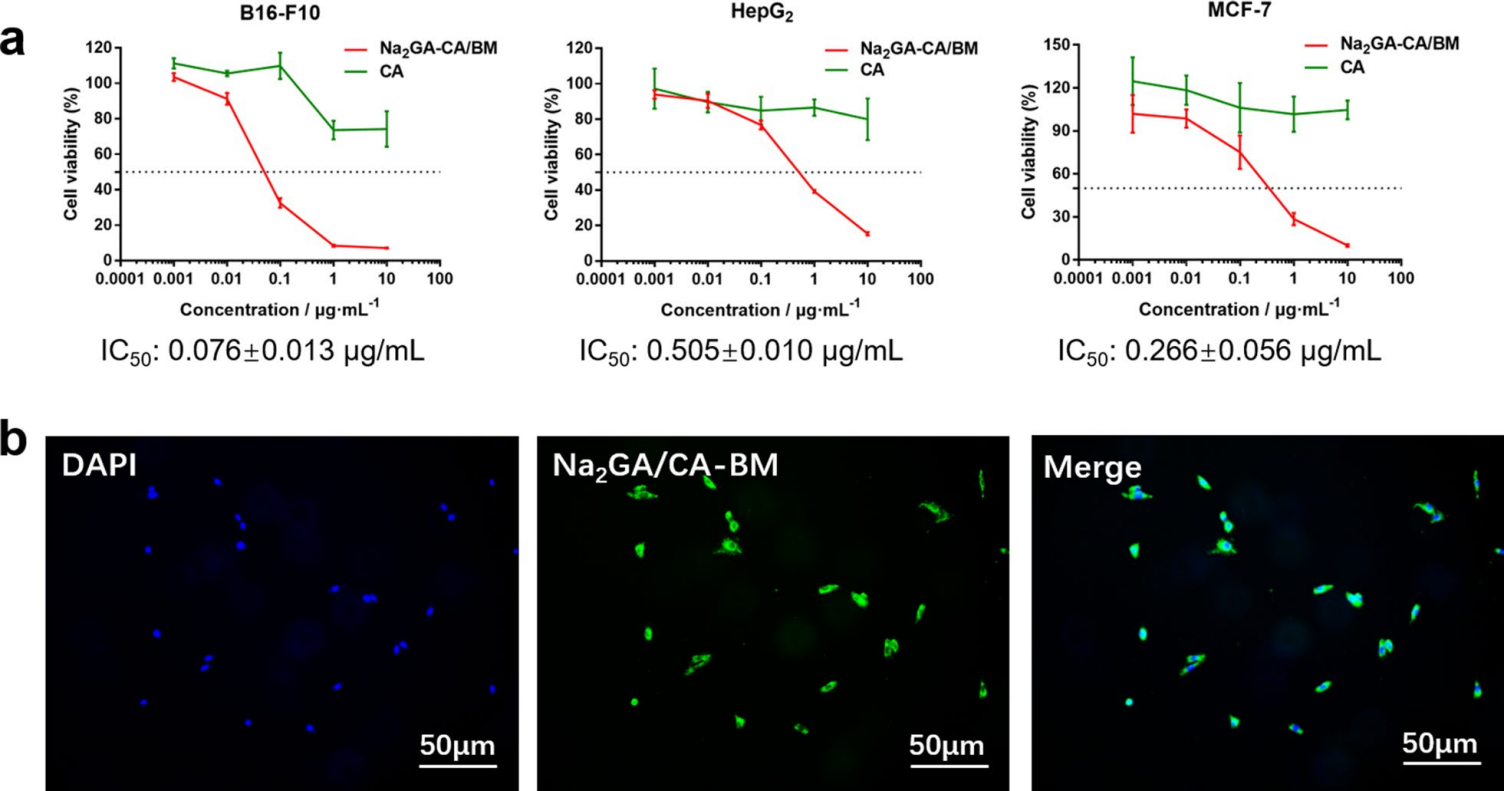
In vitro cytotoxicity and cellular uptake. **a** In vitro cytotoxicity of CA and Na_2_GA/CA-BM on B16-F10 cells, HepG_2_ cells, and MCF-7 cells, respectively (n = 3). **b** Cell uptake of Na_2_GA/CA-BM/C6. Nucleus was stained with DAPI. Images were taken from the DAPI channel (blue), Na_2_GA/CA-BM/C6 channel (green), and the overlapped image

As shown in Fig. 4a, compared with the suspension of free CA, Na_2_GA/CA-BM have the significant inhibition ability in all three kinds of tumor cells. The half maximal inhibitory concentrations (IC_50_) of Na_2_GA/CA-BM were 0.076 ± 0.013 μg/mL on B16-F10 cells, 0.505 ± 0.010 μg/mL on HepG_2_ cells, and 0.266 ± 0.056 μg/mL on MCF-7 cells, respectively. Especially, the cytotoxicity of Na_2_GA/CA-BM on B16-F10 cells was statistically significant compared with other two tumor cell lines. As shown in Table 2, the survival rate of all three cell lines was between 72 and 108.6%, which indicated that Na_2_GA itself possessed almost no cytotoxicity in all tested cell lines in the concentration range of 0.099–990 μg/mL. Therefore, Na_2_GA/CA-BM enhanced the cytotoxic ability of CA, and all obtained cytotoxic action of Na_2_GA/CA-BM was due to the CA effect. Mechanical ball milling and Na_2_GA increased the solubility of CA in water, resulting in the increase of its concentration in suspension.

**Table 2:** Cytotoxicity of Na_2_GA in B16-F10, MCF-7 and HepG_2_ cell lines

Concentration of Na_2_GA (µg/mL)	Viability (%)		
	B16-F10	MCF-7	HepG_2_
0.099	97.1 ± 9.5	100.9 ± 7.6	100.9 ± 5.2
0.99	98.0 ± 7.7	108.6 ± 12.7	105.2 ± 2.9
9.9	92.9 ± 4.4	104.2 ± 9.3	102.1 ± 2.4
99	95.1 ± 3.6	97.0 ± 19.2	98.1 ± 1.5
990	72.0 ± 6.9	73.1 ± 4.3	90.6 ± 5.3

As for the colocalization and internalization by B16-F10 cells of coumarin-6-loaded Na_2_GA/CA-BM (Na_2_GA/CA-BM/C6), the fluorescence images were shown in Fig. 4b, indicating that Na_2_GA/CA-BM/C6 was quickly taken up by B16-F10 cells and located in cytoplasm of the tumor cells.

### Pharmacokinetic evaluation

**Fig. 5:**
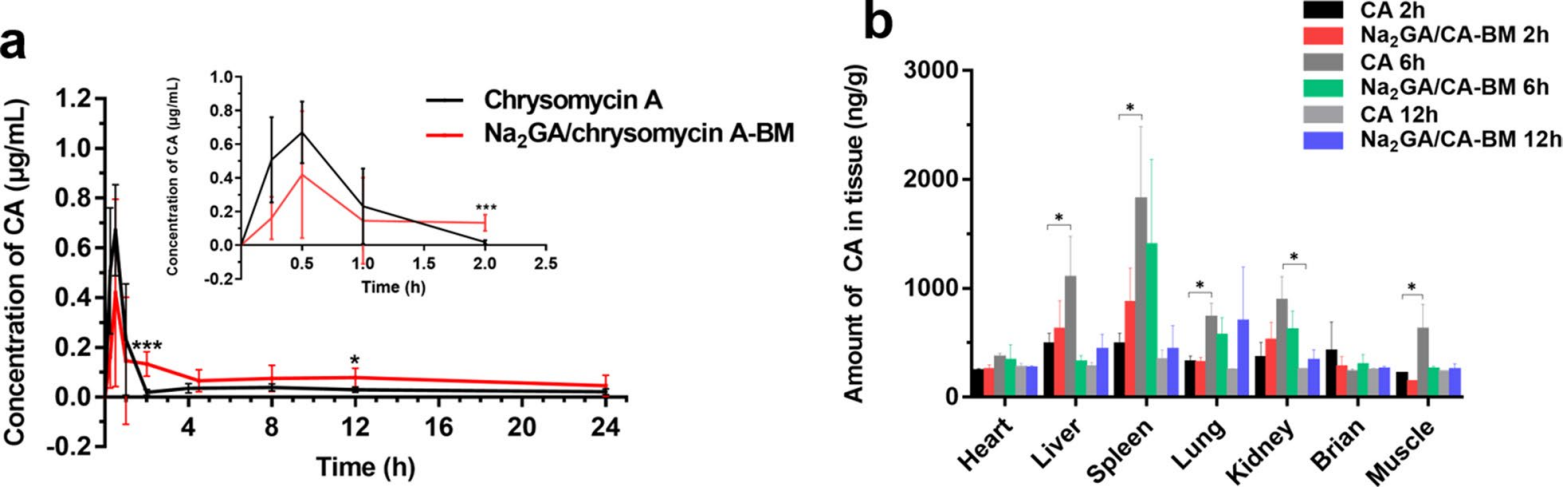
**a** Concentration of CA in ICR mice plasma after intragastric administration of two CA formulations. n = 5, *p < 0.05, ***p < 0.001. **b** Biodistribution of chrysomycin A in major tissues of mice after intragastriclly administering. n = 5, *p < 0.05

The concentration–time curves of CA in mice plasma are depicted in Fig. 5a, and the pharmacokinetic parameters are summarized in Table 3. From the figure, it could be clearly seen that the bioavailability of Na_2_GA/CA-BM was improved than pure CA. After intragastric administration, CA and Na_2_GA/CA-BM both distributed rapidly and reached the max blood concentration at 0.5 h. What’s more, the accumulation time in the body of Na_2_GA/CA-BM was longer about twofold than the retention time of free CA. Then, the free CA was cleared faster from blood than Na_2_GA/CA-BM, so Na_2_GA/CA-BM had a better blood circulation in the body. Compared with CA, the area under the curve of Na_2_GA/CA-BM was increased about 1.8 times larger, and the plasma clearance was dramatically decreased.

**Table 3:** The pharmacokinetic parameters of pure chrysomycin A and Na_2_GA/CA-BM

Samples	C_max_ (μg/mL)	T_max_ (h)	T_1/2_ (h)	AUC_0→t_ (μg/mL,·h)	AUC_0→∞_ (μg/mL,·h)	CL (L/h/kg)
CA	0.67	0.50	7.55	1.62	1.72	29.02
Na_2_GA/CA-BM	0.42	0.50	13.98	2.17	3.09	17.14

*C*_*max*_ peak plasm concentration, *T*_*max*_ time to reach peak concentration, *T*_*1*/2_ half life, *AUC* area under the plasm concentration–time curve, *CL* plasm clearance

### Tissue distribution study

Figure 5b depicts the distribution concentration of CA in major tissues of mice including heart, liver, spleen, lung, kidney, brain, skeletal muscle after oral dose of 50 mg/kg of either Na_2_GA/CA-BM or CA. After intragastric administration for 2 h, the concentration of CA (Na_2_GA/CA-BM group) was high in spleen, and reached to the highest blood concentration at 6 h, after that CA gradually cleared and finally expelled at about 12 h. As for free CA group, CA was distributed mainly in spleen, liver, lung, kidney and muscle, and slowly cleared after 12 h. The main metabolic organ of CA was liver and spleen, while Na_2_GA/CA-BM was metabolized mainly in spleen after 6 h. Generally, large size of particles were preferentially absorbed by the liver, and small particles were easily cleared by the spleen, which led to the change in metabolic site of drug [36, 37]. In comparison, Na_2_GA/CA-BM showed longer blood accumulation in body than free CA after 12 h, which was consistent with the results of the pharmacokinetic study.

### In vivo antitumor efficacy

**Fig. 6:**
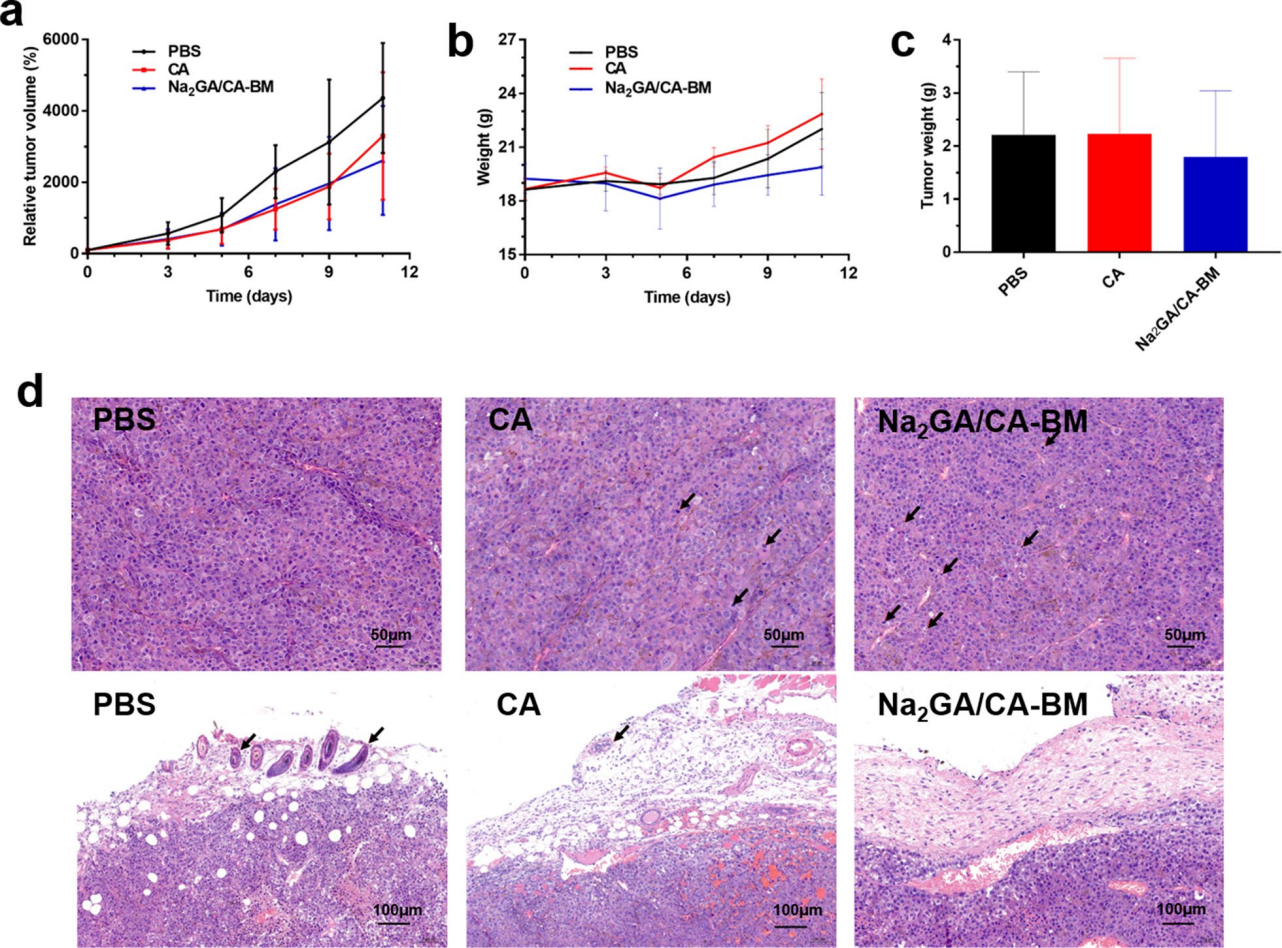
In vivo antitumor effect test in C57BL/6 mice by intragastric administration (n = 5). **a** The change curve of tumor volume throughout the treatment. **b** The body weight curve of mice. **c** Mean weight of tumor in each group at the end of treatment. **d** H&E staining of tumor tissue sections with envelop layer

Due to the better performance on the solubility and bioavailability, we next evaluated the antitumor ability of Na_2_GA/CA-BM on B16-F10 tumor-bearing C57BL/6 mice. As shown in Fig. 6a, CA and Na_2_GA/CA-BM both inhibited the growth of B16-F10 tumors compared with the control group. Meanwhile, Na_2_GA/CA-BM showed better tumor suppression ability throughout the treatment, and the tumor inhibition rate was closed 28.76%. From Fig. 6b, it was found that none of the mice loss body weight obviously after treating CA formulations, which indicated no potential systemic toxicities of CA and Na_2_GA/CA-BM.

After tumors excision, the weights (Fig. 6c) of tumors treated with Na_2_GA/CA-BM (1.800 ± 1.246 g) were lower than those of the mice treated with CA (2.230 ± 1.429 g) or PBS (2.212 ± 1.192 g). In addition, the tumor weights of CA group showed no significant differences compared with the control group.

H&E staining were shown to observe pathological changes of tumor cells in three groups, As shown in Fig. 6d, the tumor cells were dense and had abundant vascular tissue in all three groups. Karyopycnosis and deep staining could be seen, which meant apoptosis and necrosis of cancer cells. Thus, different degrees of apoptosis were seen in tumors treated with CA and Na_2_GA/CA-BM. In comparison, a large amount of excessive vacuolization and more apoptosis cells were observed in the tissue section of Na_2_GA/CA-BM group. Moreover, various size tumor cells could be seen in the envelop layer of tumor tissue treated with PBS and the free CA, rather than in tumor layer of Na_2_GA/CA-BM group, indicating the excellent antitumor ability of Na_2_GA/CA-BM.

## Conclusions

In the present study, an amorphous CA solid dispersion was successfully prepared by mechanical ball milling. As compared to the free CA, Na_2_GA/CA-BM exhibited superior solubility evidenced by a about 50-fold increase. The physicochemical characteristics analysis showed that CA was dispersed uniformly in the hydrophilic carrier (Na_2_GA) and transformed from crystals into amorphous state by ball milling. When Na_2_GA/CA-BM dissolved in water, CA encapsulated by Na_2_GA was self-formed to micelles. Consistent with the amorphous nature and self-formed micelles of Na_2_GA/CA-BM, it showed significant improvement of pharmacokinetic behavior in mice, which increased 1.8 times in oral bioavailability. Moreover, Na_2_GA/CA-BM also exhibited a stronger antitumor ability than CA due to the improvement of oral bioavailability. In summary, our work illustrated an unprecedented and environment-friendly preparation of the CA formulation by ball milling approach, which are promising to enhance the oral bioavailability and antitumor ability of CA, might be considered for efficient anticancer therapy.

## Data Availability

The datasets used and/or analysed during the current study are available from the corresponding author on reasonable request.
